# Chlorpromazine-Induced Hyperprolactinemia on Rat’s Uterus

**DOI:** 10.7508/ibj.2015.04.006

**Published:** 2015-10

**Authors:** Zahra Zamani, Samad Zare, Rajabali Sadrkhanlou, Abbas Ahmadi, Elham Movahed

**Affiliations:** 1 *Dept. of Biology, Faculty of Science, Urmia University, Urmia, Iran; *; 2 *Laboratory of Embryology, Dept. of Basic Science, Faculty of Veterinary Medicine, Urmia University, Urmia, Iran*

**Keywords:** Hyperprolactinemia, Uterus, Rats

## Abstract

**Background:**

Hyperprolactinemia is a common side effect of antipsychotic drugs that requires further investigation. The current study was designed to evaluate dose-dependent effect of chlorpromazine (CPZ) on hormonal changes and uterine horn histological structure in rats. Moreover, the mammary glands were analyzed to show hyperprolactinemia-induced histological changes.

**Methods:**

Albino Wistar rats (n = 32) were divided into four groups. The first group was set as a control. In the three drug-treated groups (eight rats in each group), CPZ was administered by a gavage at doses of 3, 10, and 30 mg/kg/day for 28 days. One day after the last administration of the drug, the animals were sacrificed. Histopathological and histomorphometrical analyses of the uterine horns and mammary glands were carried out to evaluate dose-dependent effect of CPZ on histological structure. Serum levels of prolactin (PRL), estradiol, progesterone, follicle-stimulating hormone (FSH), and luteinizing hormone (LH) were also evaluated.

**Results:**

Remarkable (*P* < 0.05) elevation was observed in CPZ-administrated animals' uterine horn endometrium, myometrium, and perimetrium thicknesses, and the mammary glands were observed with galactorrhea features. The serum level of progesterone and PRL significantly (*P* < 0.05) increased, while the serum concentration of LH, FSH, and estradiol was notably (*P* < 0.05) decreased depending on administrated CPZ dose. No histological and biological changes were occurred in the control animals.

**Conclusion:**

The present findings suggest that CPZ-induced disturbances not only depend on PRL level and increased PRL level largely depends on administrated doses of the CPZ.

## INTRODUCTION

Chlorpromazine (CPZ) is an antipsychotic drug that selectively blocks postsynaptic dopaminergic neurons [1]. This compound affects D2 receptors and is widely used to treat schizophrenia as an antipsychotic agent [2].

All antipsychotic medications are associated with increased risk of sexual dysfunction, cardiac arrhythmia, postural hypotension, and sudden cardiac death [3, 4]. Understanding the profiles adverse effects of these medications is necessary for treatment of the schizophrenia patients. The common side effect of antipsychotic treatments is the increase of prolactin (PRL) level (hyperprolactinaemia) that has received a little attention [5]. Antipsychotic drugs block dopamine D2 receptors on lactotroph cells in the anterior pituitary gland and remove the inhibitory influence on PRL secretion [2, 6]. Researchers have shown that hyperprolactinemia has impacts on fertility and sexual performance [7].

Gonadotropin-releasing hormone (GnRH) directly affects the pituitary physiologic functions and causes gonadotropins (luteinizing hormone [LH] and follicle-stimulating hormone [FSH]). PRL suppresses GnRH secretion from the hypothalamus [8]. Moreover, physiological function of follicular growth and granulosa cells mainly depends on the serum level of FSH and LH [9]. Thus, the dysregulation of ovarian hormones would lead to important problems in fertilizing potential [9]. Disorder in gonadotropines function is related to the pituitary gland and its feedback mechanisms. Antipsychotic treatment is often initiated when patients are in their late teens or twenties and continues for years or decades [10], and women in reproductive age are influenced by the medical disorders associated with hyperprolactinemia. 

Thus, the present study was designed to evaluate the dose-dependent effect of CPZ on hormonal changes and uterine horn histological structure in rats. Moreover, the mammary glands were analyzed to show the hyperprolactinemia-induced histological changes.

## MATERIALS AND METHODS

***Animals.*** Female Wistar rats (n = 32, 70 days old, 160 ± 5 g) were obtained from Animal House of Faculty of Science, Urmia University, Iran and were acclimatized in an environmentally controlled room with 12h light/12h dark and temperature of 22 ± 2ºC. A standard pellet food and tap water were available *ad libitum*. In this study, all experiments conducted on animals were in concord with the Urmia University guidance of ethical committee for research on laboratory animals. Animals were allowed to acclimate for one week before experimental use.

***Drug treatment*** ***.*** CPZ (Sigma-Aldrich Co., Germany) was used at three dose levels (3, 10, and 30 mg/kg/day) based on a previous study [11], and then dissolved in 0.5% methyl cellulose solution [12]. The administer-ation of the drug to the female rats was carried out by an oral gavage. Following a week acclimation, the animals were assigned into four groups of eight rats. One group was selected as control, and 5 ml/kg of 0.5% methyl cellulose solution was administrated once a day for 28 consecutive days. Three groups were chosen as test groups, and CPZ was administered 3, 10, and 30 mg/kg/day for 28 consecutive days.

***Hormonal assay.*** At 29 days, the animals from each group were sacrificed by CO_2_ inhalation, and the blood samples were collected from the jugular vein. PRL is a stress hormone; therefore, for evaluating PRL levels without stress effects, we chose rapid decapitation, which is actually far less stressful for the rodent. Also, to avoid smelling the blood of other rats, the animals were scarified separately. Blood sera were separated by centrifugation (3000 ×g, at room temperature for 5 min), and subjected to the assessment of the serum level of PRL, LH, FSH, progesterone, and estrogen.

***Radioimmunoassay of*** ***PRL, LH, and FSH in serum. ***Sera (100 μl) were added to the tubes containing 100 μl labeled hormones with the rabbit antisera in 0.01 M phosphate buffer at pH 7.6. Anti-rat PRL (Cisbio Bioassays, France) as well as FSH and LH were diluted 1:5000, 1:2500, and 1:10,000, respectively. The diluted goat anti-rabbit IgG (1:10, 200 μl) was then added to the mixture. After allowing to stand for 18 h at 40ºC, the mixture was centrifuged at 2000 ×g at room temperature for 30 min, and radioactivity in the resulting pellets was measured in a gamma counter. 

***Radioimmunoassay of serum estradiol and progesterone. ***Concentrations of serum estradiol were measured by using CIS kits (Cisbio Bioassays, France) in accordance with the methods given by the manufacturer. Serum (300 μl) was extracted with 3 ml ethyl ether. The layer of ether was evaporated under N2 gas, and the extract was resuspended in 300 μl 0.04 M phosphate buffer. After the addition of 100 μ1 17/3-estradiol (14,000 CPM), each tube was incubated with 100, µl antiserum raised in rabbits at room temperature for 18 h. Next, goat anti-rabbit r-globulin (1 ml) was added, and the mixture was incubated at room temperature for 15 min. Following centrifugation, the radioactivity in the pellet was counted. To evaluate the serum level of progesterone, a mixture of ethylether (1 ml) and propylene glycol (50 μ1) was prepared. After evaporation of ether under N_2_ gas, 0.5 ml phosphate buffer and 0.1 ml (20,000 CPM) iodo progesterone were added, and the mixture was incubated with 0.1 ml antiserum raised in rabbits at room temperature for 18 h. Afterward, 0.1 ml bovine serum gamma globulin and polyethylene glycol were added to the mixture, followed by centrifugation at 2000 ×g at room temperature for 10 min. Finally, the radioactivity was measured in the pellet [13].

***Histology and morphometry. ***The specimens from uterine horn and mammary gland were dissected out and fixed in 10% formalin fixative for histological analysis and subsequently embedded in paraffin. The prepared sections (5-6 μm) were stained with hematoxylin-eosin (Merck Co., Germany) staining. For histomorphometric analysis, the thickness of uterine horns endometrial epithelium, endometrium, myometrium, and perimetrium, the diameter of the mammary glands lobules, and histological features were evaluated by a morphometric lens (magnification 40×, Olympus, Germany). The gland distribution and the number of the gland per one mm^2^ of endometrium were estimated. For morphometric analysis, the stained sections were observed by using the eyepiece scale (occulometer) and the stage micrometer. The eye piece occulometer was divided into two 100 small divisions. The stage micrometer scale was made up to 1 mm divided into 0.1 mm division. Each 0.1 mm was divided into 0.01 mm and the eye piece scale (occulometer) was inserted into the eye piece of the microscope. Next, the eye piece scales and the stage were adjusted until there was a parallel point between the two scales and the number of the eye piece divisions, and its corresponding stage measurements was noted; if 70 occulometer divisions was equal to 14 μm, all the objective lens were thus calibrated. The occulometer was fixed into the Olympus Microscope and focused through stained sections of the tissue to allow for the measurement of the parameters [14]. Follicular and corpora lutea (CL) morphology was examined by a microscope under 40× objective lens (Olympus, Germany). Follicles with a complete layer of flattened granulosa cells, a normal nucleus, and oocytes with cytoplasm were considered as normal follicles. Abnormal follicles were classified as follows: pyknotic nucleus, cytoplasmic damage, and combination of damaged nucleus and cytoplasm. For CL analysis, CLs sizes in treatment groups were compared with those in the control [15]. 

**Table 1:** Mean serum levels of prolactin, luteinizing hormone (LH), follicle-stimulating hormone (FSH), estrogen and progesterone in different groups

Hormones	Control	3 mg/kg	10 mg/kg	30 mg/kg
Prolactin (ng/ml)	55.75 ± 3.06	109.25 ± 13.37	223.75 ± 26.35^ab^	249.50 ± 25.82abc
LH (ng/ml)	0.56 ±0.05	0.58 ±0.06	0.30 ±0.02^ab^	0.26 ± 0.02^ab^
FSH	3.17 ± 0.48	1.97± 0.44	1.35 ± 0.2 ab	1.13 ± 0.06^ab^
Estrogen (pg/ml)	41.50 ± 2.62	29.00 ± 1.47	29.50± 2.59^ab^	24.00 ± 0.40^ab^
Progestron (ng/ml)	18.12 ± 2.55	22.75 ± 3.11	32.07 ±3.75^ab^	33.82± 3.71^ab^

a,b,c indicate significant differences (*P* < 0.05) between data of CPZ-administrated groups with control group, 3 mg/kg, and 10 mg/kg, respectively. All data are presented as mean ± SD. Data indicate significant differences (*P* < 0.05) between CPZ groups (n = 8 for each group) and the control group (n = 8 for each group).

***Statistical analysis. ***The data were expressed as the mean ± SD. Experimental data were analyzed by using the analysis of variance and Duncan's multiple range test (SPSS, 16.00, Chicago, Illinois, USA).

## RESULTS

***Hormone*** ***concentrations. ***Biochemical analysis showed that the serum levels of PRL were significantly (*P* < 0.05) increased in CPZ-administrated animals, which were dependent on CPZ administration doses. On the contrary, in control animals, the PRL level was constant. The analysis of the serum levels of FSH and LH between test and control groups showed remarkably significant (*P* < 0.05) decrease in CPZ-induced animals; this reduction in FSH and LH levels progresses regarding CPZ administration doses. The progesterone level was remarkably (*P* < 0.05) increased while the serum levels of estradiol were significantly (*P* < 0.05) decreased in CPZ-received animals, which depends on CPZ administration doses. The data for hormonal analysis are presented in Table 1.

**Fig. 1:**
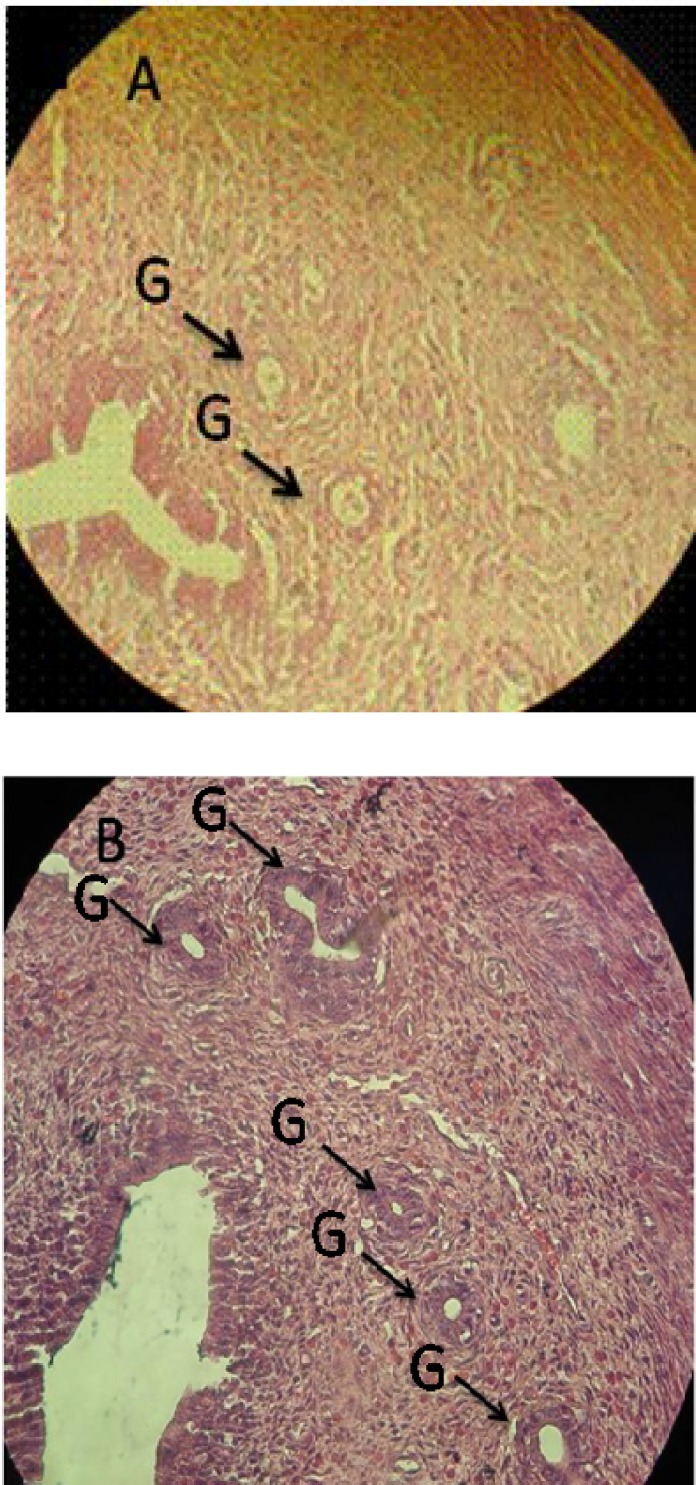
Uterine horn cross-section. (A) control group, normal gland distribution in uterine horn; (B) High dose CPZ group (30 mg.kg). The gland distribution significantly increased per 1 mm^2^ of the endometrium in comparison to other groups (hematoxylin-eosin staining, magnification 400×). G, glands (arrows

**Fig. 2:**
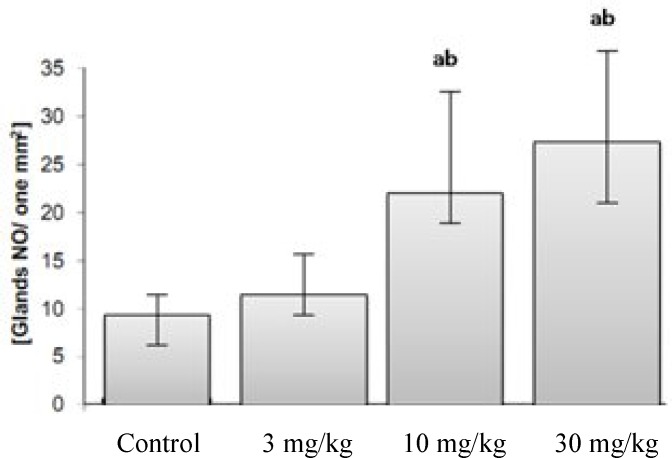
Mean distribution of the glands per 1 mm^2^ of the uterine endometrium in different groups. CPZ administration increased gland number in endometrium. ^a,b ^indicate significant differences (*P* < 0.05) between data of CPZ-administrated groups with control group, 3 mg/kg, and 10 mg/kg, respectively. All data are presented as mean ± SD.

***Histomorphological results.*** The comparison of the uterine horn endometrial epithelium height (left and right) among all groups showed that in CPZ-administrated groups, the epithelial height was significantly (*P* < 0.05) increased compared to the control animals. This impairment was developed depending on the dose of the CPZ. Accordingly, the high dose administrated group of CPZ showed the highest endometrial epithelium. Simultaneously, the perimetrium, myometrium, and endometrial thicknesses in both sides of uterine horns were remarkable (*P* < 0.05) increased with high doses of CPZ-administrated groups (Table 2). Analyzing the endometrial glands numbers per one mm^2^ of the endometrium revealed that in CPZ-received groups, the gland distribution was considerably (*P* < 0.05) increased in number per one mm^2^ of the endometrium. Meanwhile, the animals in the control group were shown with remarkably lower numbers of endometrial glands per mm^2^ of the endometrium (Figs. 1 and 2).

**Fig. 3:**
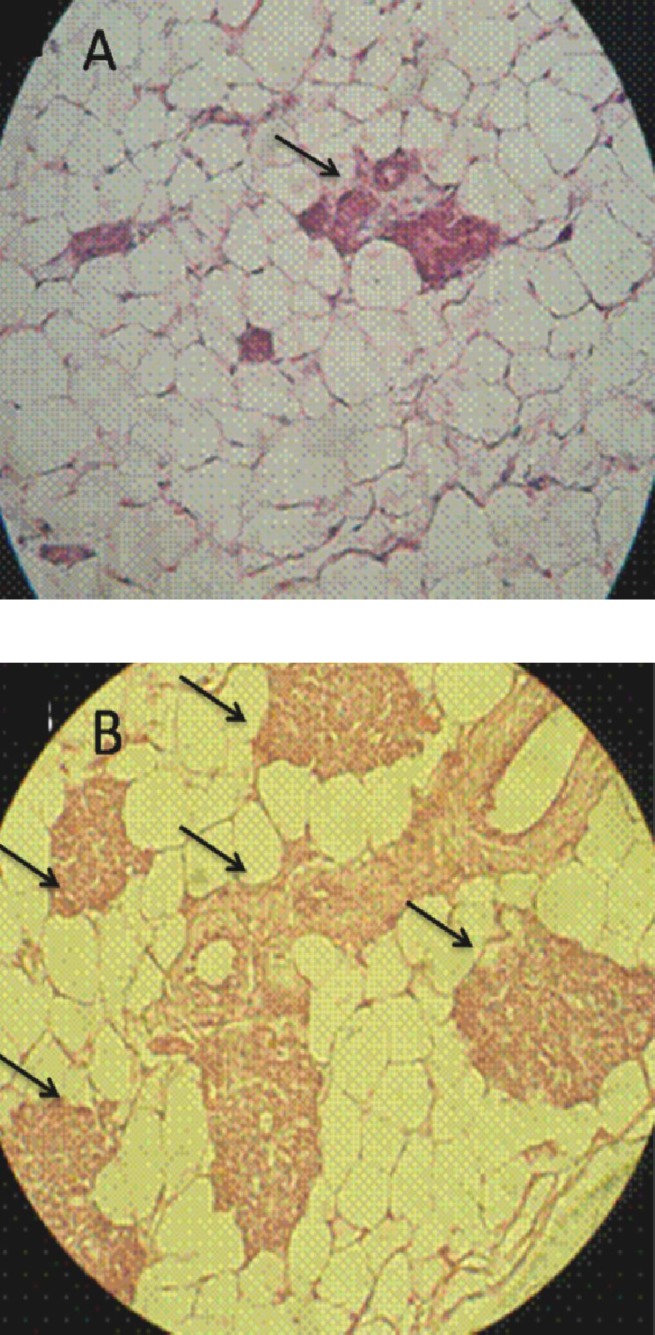
Mammary gland cross-section. Note inactive mammary glands in control (A). Developed secretory alveoli and ducts are presented in high dose (B) in CPZ-administrated groups. The lactating alveolus diameter in glands and the lactiferous duct distribution considerably was increased in CPZ-administrated animals (hematoxylin-eosin staining, magnific-ation 400×). Arrows indicate lactating alveolus

Light microscopic analysis indicated that the lactating alveolus diameter in glands and the lactiferous duct distribution were remarkably (*P* < 0.05) increased in CPZ-administrated groups. Also, the alveolar epithelium height was significantly increased in CPZ-received animals. This situation progressed depending on CPZ-administrated dose. Our histology-cal analysis demonstrated that the fatty globules synchronized with remarkable secretory alveoli and duct development were manifested in secretory and intra lobular ducts of the CPZ- administrated rat mammary glands (Fig. 3). However, the glands of the control animals were manifested with inactive lobules and secretory ducts. The data for histomorphometric analysis are indicated in Table 3.

**Table 2:** Histomorphometric data for uterine horns in different groups

**Parameters (μm)**	**Control**	**3 mg/kg**	**10 mg/kg**	**30 mg/kg**
Uterine horn diameter	1241.02 ± 102.97	1539.65 ± 21.98	1780.25 ± 107.02^a^	2296.28 ± 119.59^ab^
Endometrial epithelium height	12.53 ± 2.50	20.06 ± 2.89	25.05 ± 4.09	42.60 ± 4.79^ab^
Endometrial thickness	265.67± 28.85	358.21± 11.69	368.37 ±28.41	426.50 ±29.90 a
Myometrium thickness	205.31 ± 43.12	287.32 ± 34.35	340.21 ± 42.25^a^	517.25± 56.31^ab^
Perimetrium thickness	16.25 ± 2.89	26.50 ± 3.06	38.50 ± 5.60^a^	41.56 ±4.79^a^

a,b indicate significant differences (*P* < 0.05) between data of CPZ-administrated groups with control group, 3 mg/kg, and 10 mg/kg, respectively. All data are presented as mean ± SD. CPZ administration increased uterine horn diameter by elevating endometrial, myometrium, and perimetrium thicknesses

**Fig. 4:**
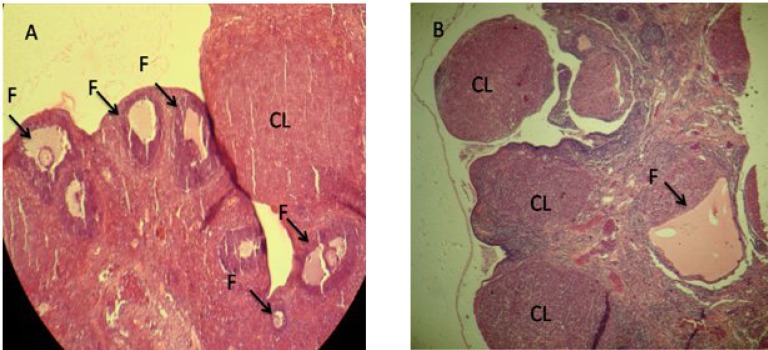
Ovary cross-section. (A) Control group: the ovary from the control group is presented with different size follicles (F) and corpora lutea (CL); (B) The highest dose CPZ treatment group (30 mg/kg): the ovary from the treatment group in the highest dose of CPZ is presented with large and active CL without follicular growth (hematoxylin-eosin staining, magnification 400×)

Histological analysis in this study showed that in CPZ-administrated groups, the normal follicles were significantly decreased in comparison to the control animals. In test groups, atretic follicles were higher than control, which depends on CPZ administration doses (Fig. 4). Moreover, observations demonstrated that CPZ-administrated animals in two high doses exhibited significantly higher sizes of CLs in comparison to the control group (Fig. 4). 

## DISCUSSION

In the present study, CPZ (an antipsychotic drug) was used to analyze the effect of CPZ-induced hyperprolactinemia on reproductive system and functions in female rats mediated via the hypothalamic-pituitary-gonadal system. Although hormonal analysis demonstrated the increased levels of serum PRL and progesterone, the serum levels of estrogen, FSH, and LH in rats was decreased by the administration of CPZ in a dose-dependent manner. Moreover, histological examination showed a significant increase in uterine horn wall thickness, and the mammary glands were observed with galactorrhea features. Furthermore, CPZ significantly increased the atretic follicle formation and also exceeded the size of normal CL in two high doses. In a previous study, it has been demonstrated that the number of atretic follicles formation, the rate of normal follicles, and the size of normal CL have been increased [16]. Our study has indicated a similar outcome. Some surveys, have reported that dopamine plays a crucial role in tonic inhibition of PRL secretion [17, 18] and inhibits PRL secretion while it affects lactotroph cells in the anterior pituitary gland [19]. In one study, the use of an antipsychotic drug, haloperidol, to inhibit dopamine secretion resulted in the increased PRL level in rats [18]. The results from biochemical analysis in the present study corroborate the mentioned hypothesis and accordingly the serum levels of PRL was significantly (*P* < 0.05) increased in CPZ-administrated groups in a dose-dependent manner.

**Table 3:** Histomorphometric data for mammary gland lobules in different group

Parameters (μm)	Control	3 mg/kg	10 mg/kg	30 mg/kg
Lobular diameter	48.75 ± 4.43	84.12 ± 7.15	185.65 ± 54.80^ab^	254.03 ± 34.23abc

a,b,c indicate significant differences (*P* < 0.05) between data of CPZ-administrated groups with control group, 3 mg/kg, and 10 mg/kg, respectively. All data are presented as mean ± SD. CPZ administration increased the lobular diameter in a dose-dependent manner.

It is well established that PRL can prevent luteolysis and cause increased numbers of persisting CL [20]. Also, some studies have reported that the high levels of PRL inhibit the secretion of GnRH from the hypothalamus axis [21, 22]. The pulsatile secretion pattern of GnRH causes the cyclic release of LH and FSH, and the inhibition of GnRH results in the reduction of LH and FSH [23]. In mammals, FSH induces follicle growth and subsequently estradiol secretion by the granulose cells [24, 25]. Here, we showed that the serum level of estrogen was decreased, whereas the progesterone concentration was increased in CPZ-administrated groups, which are dependent on doses in our study. These abnormalities may be marked by higher levels of PRL. PRL caused resistant CL from previous cycles (in turn led to severe follicular atresia), and this CLs did not allow the estradiol secretion to restart. The decreased serum level of estrogen in CPZ-administrated animals approved the mentioned theory. 

As mentioned above, the increased level of PRL largely can affect gonadotropins. Our analysis showed that the serum levels of LH and FSH were significantly decreased in two groups that received the high doses of the CPZ. In patients treated with antipsychotic drugs, the reduced secretion of the GnRH in the hypothalamus was able to decrease stimulation for LH and FSH secretion in the pituitary gland [26]. Thus, we can conclude that the CPZ with direct and indirect hyperprolactinemia blocks the hypothalamus-pituitary axis that in turn inhibited the gonadotropins secretion. Additionally, the estrogen positive feedback in the pituitary gland for LH hormone secretion was eliminated. Therefore, the serum levels of LH and FSH was significantly decreased in CPZ-administrated animals. Also, the decreased estrogen level related to reduced gonadotropins. Reduction in gonadotropin levels resulted in CL resistance that was delivered from the previous cycle, and ultimately occurred situation increased atresia in CPZ-administrated animals. The biological activity of the CLs demonstrates with serum level of progesterone cycle [27]. Our observations showed that the serum level of progesterone was significantly increased in animals consumed CPZ, indicating increased function of CLs. During the estrous cycle, the level of estrogen increases at proestrus and becomes low during estrus, metestrus, and diestrus [28]. Therefore, in our study, lower serum estrogen level in the treated animals was consistent with the persistence of the diestrus phase in these animals [28]. PRL has a luteotropic activity and causes stimulating and maintaining the CL in rodents. Because of this effect of PRL, hyperprolactinemia is known to be one of the causes of pseudopregnancy, namely continuous diestrus [16].

Light microscopic observations demonstrated that the endometrial thickness and the glandular structure of the endometrium were significantly increased by administrating the high doses of CPZ groups. With regard to the luteotrophic effect of PRL and at the same time the increase in progesterone level, we can hypothesize that following the increased level of PRL and accomplishing this impairment with higher concentration of progesterone, the endometrial thickness and gland distribution are increased in CPZ-administrated animals. Accordingly, in administration of CPZ in high doses, the number of glands per one mm^2^ was remarkably increased. Alveolar development of mammary glands as well as alveolar epithelium proliferation and differentiation are dependent on the PRL hormone stimulation [29]. Histological analysis manifested the fatty globules in secretory and intra lobular ducts mammary glands in high doses CPZ-administrated rats. These features show that the severity of galactorrhea mainly depends on the PRL level, which was considerably increased in the high doses CPZ -administrated group.

Our results indicated that although CPZ is widely used as an antipsychotic drug, the rats treated with CPZ were found to have mean serum PRL levels that may be several-fold greater than the upper limit of normal. Additionally, the CPZ-induced hyper-prolactinemia was associated with a disturbance in the levels of essential reproductive hormones estradiol and progesterone. Moreover, the PRL-associated abnormalities in gonadotropins and reproductive hormones exerted a significant effect on galactorrhea features and thickness of the epithelium, myomterium and endometrium of the uterus in CPZ-administrated rats. 

## References

[1] Peuskens J, Pani L, Detraux J, De Hert M (2014). The effects of novel and newly approved antipsychotics on serum prolactin levels. CNS Drugs.

[2] Bargiota S, Bonotis K, Messinis I, Angelopoulos N (2013). The effects of antipsychotics on prolactin levels and women’s menstruation. Schizophr Res.

[3] Inder WJ, Castle D (2011). Antipsychotic-induced hyperprola ctinaemia. Aust N Z J Psychiatry.

[4] Wu H, Deng L, Zhao L, Zhao J, Li L, Chen J (2013). Osteoporosis associated with antipsychotic treatment in schizophrenia. Int J Endocrinol.

[5] Ochoa S, Usall J, Cobo J, Labad X, Kulkarni J (2012). Gender differences in schizophrenia and first-episode psychosis. Schizophr Res.

[6] Bains S, Shah A (2012). Sexual side effects of antipsychotic drugs. Pharmacoepidem Drug Safety.

[7] Wasiu Eniola O, Adebambo Olufemi A, Adebayo Adetola A, Ademola Oladipupo M (2012). Pattern of reproductive hormones (follicle stimulating hormone, luteinizing hormone, estradiol, progesterone, and prolactin) levels in infertile women in Sagamu South Western Nigeria. Der Pharmacia Letter.

[8] Laway B, Mir S (2013). Pregnancy and pituitary disorders: Challenges in diagnosis and management. Indian J Endoc Met.

[9] Kishimoto T, Hert M, Carlson H, Manu P, Correll C (2012). Osteoporosis and fracture risk in people with Schizophrenia. Curr Opin Psychiatry.

[10] Liu C, Demjaha A (2013). Antipsychotic interventions in prodromal psychosis. CNS Drugs.

[11] Izumi Y, Watanabe T, Awasaki N, Hikawa K, Minagi T, Chatani F (2008). Collaborative work on evaluation of ovarian toxicity. Effects of 2 or 4 weeks repeated dose studies and fertility study of chlorpromazine hydrochloride in rats. J Toxicol Sci.

[12] Kunimatsu T, Kimura J, Funabashi H, Inoue T, Seki T (2010). The antipsychotics haloperidol and chlorpromazine increase bone metabolism and induce osteopenia in female rats. Regul Toxicol Pharm.

[13] Othman A, Jesse FA, Adamu L, Abba Y, Adza Rina M, Saharee A (2014). Changes in serum progesterone and estrogen concentrations in non-pregnant boer does following experimental infection with Coryneba-cterium pseudotuberculosis. J Vet Adv.

[14] Chatterjee A, Chatterji U (2010). Arsenic abrogates the estrogen-signaling pathway in the rat uterus. Reprod Biol Endocrinol.

[15] Khoram H, Najafpour A, Razi M (2011). Follicular viability and histological alterations after auto-transplantation of dog ovaries by experimentally inducing blood sinus on stomach. Int J Fertil Stril.

[16] Egli M, Leeners B, Kruger T (2010). Prolactin secretion patterns: basic mechanisms and clinical implications for reproduction. Reproduction.

[17] Bargiota S, Bonotis K, Messinis I, Angelopoulos N (2013). The effects of antipsychotics on prolactin levelsand women’s menstruation. Schizophr Res Treat.

[18] Koroglu A, Hocaoglu C (2012). Risperidone-induced acromegaly: a case report. Ther Adv Psychopharmacol.

[19] Kelly D, Wehring H, Earl A, Sullivan A, Dickerson F, Feldman S (2013). Treating symptomatic hyperpr-olactinemia in women with schizophrenia: presentation of the ongoing DAAMSEL clinical trial (dopamine partial agonist, aripiprazole, for the management of symptomatic elevated prolactin). BMC Psychiatry.

[20] Bazer F, Song G, Thatcher W (2012). Roles of conceptus secretory proteins in establishment and maintenance of pregnancy in ruminants. Asian-Australas J Anim Sci.

[21] Assidi M, Richard F, Sirard A (2013). FSH in vitro versus LH in vivo: similar genomic effects on the cumulus. J Ova Res.

[22] Zandi M, Jafarzadeh Shirazi M, Tamadon A, Akhlaghi A, Salehi M, Niazi A (2014). Hypothalamic expression of melanocortin-4 receptor and agouti-related peptide mRNAs during the estrous cycle of rats. Int J Mol Cell Med.

[23] Clarke I (2011). Control of GnRH secretion: One step back. Front Neuroendocrinol.

[24] Peretz J, Craig Z, Flaws J, Bisphenol A (2012). Inhibits follicle growth and induces atresia in cultured mouse antral follicles independently of the genomic estrogenic pathway. Biol Reprod.

[25] Wang Q, Leader A, Tsang B (2013). Follicular stage-dependent regulation of apoptosis and steroidogenesis by prohibitin in rat granulosa cells. J Ova Res.

[26] McGuire N, Koh A, Bentley G (2013). The direct response of the gonads to cues of stress in a temperate songbird species is season-dependent. Peer J.

[27] Mohammadi Khanghah K, Moradi kor N (2013). A review of biology and function of corpus luteum. J Biol Today's world.

[28] Lueders I, Taya K, Watanabe G, Yamamoto Y, Yamamoto T, Kaewmanee S (2011). Role of the double luteinizing hormone peak, luteinizing follicles, and the secretionof inhibin for dominant follicle selection in Asian elephants (Elephas maximus). Biol Reprod.

[29] Sakamoto K, Triplett A, Schuler L, Wagner K (2010). Janus kinase 2 is required for the initiation but not maintenance of prolactin-induced mammary cancer. Oncogene.

